# 2D Tellurene‐Based Optoelectronic Memristor with Temporal Dynamics for Multimodal Reservoir Computing System

**DOI:** 10.1002/advs.202513647

**Published:** 2025-09-04

**Authors:** Jingyao Bian, Zhuangzhuang Li, Shuang Liang, Ye Tao, Zhongqiang Wang, Yongxing Zhu, Changhua Wang, Haiyang Xu, Yichun Liu

**Affiliations:** ^1^ State Key Laboratory of Integrated Optoelectronics Key Laboratory of UV Light‐Emitting Materials and Technology of Ministry of Education Northeast Normal University Changchun Jilin 130024 China; ^2^ School of Science Heilongjiang University of Science and Technology Harbin 150020 China

**Keywords:** 2D materials, neuromorphic perception system, optoelectronic memristor, solution plasma process, tellurene

## Abstract

Neuromorphic multimodal perception of sensory systems can integrate the stimulation from different senses, thus enhancing the perception accuracy of organisms to understand the external environment. An optoelectronic memristor with the capability to combine multidimensional sensing and processing functions is highly desirable for developing efficient neuromorphic multimodal sensory systems (MSSs). In this work, a tellurene (Te) nanoflake‐based optoelectronic memristor relying on solution plasma process (SPP) treatment is demonstrated for the first time, which is capable of combining infrared (IR) optical and electrical stimuli in a single synaptic device for a multisensory integration function. The multimode switching mechanisms can be attributed to the migration of SPP‐induced defects under an electric field and the charge trapping/detrapping in those defects under light illumination. Furthermore, a multimode reservoir computing (MRC) system is realized to perform pattern recognition tasks using the IR optical (1022 nm) and electrical stimulations, including the fusion of visual and haptic multimode sensory and physiological recognition tasks. The accuracy of pattern recognition using MRC is obviously enhanced compared to that of individual sensory. This work provides a new approach to developing multimodal 2D Te‐based MRC for the construction of highly efficient neuromorphic MSSs.

## Graphical abstract

The SPP‐treated Te‐based optoelectronic memristor demonstrates multimodal synaptic function in a single device under infrared optical and electrical stimuli. The multimode RC system is adept for processing sequence signals, including multisensory fusion and ECG pattern recognition. A feasible way is provided to fabricate Te‐based optoelectronic memristor through SPP treatment, which is promised to realize high‐precision multimodal perception systems.

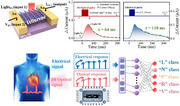

## 1 Introduction

Neuromorphic multimodal perception, as an essential function of biological sensory systems, can integrate sensory signals acquired from different senses, e.g., visual and haptic signals. This capability allows the human brain to respond to complex situations more swiftly and precisely.^[^ ^1^, ^2^, ^3^ ^]^ Therefore, artificial multimodal sensory systems (MSSs) with perceptual learning present significant potential for application in the development of autonomous soft robots, prostheses, human‐machine interfaces, and artificial intelligence technologies.^[^ ^4^, ^5^, ^6^ ^]^ In order to achieve the artificial MSSs, the circuit design containing diverse advanced sensors is usually necessary, as reported in recent literature.^[^ ^7^, ^8^, ^9^, ^10^ ^]^ For example, Tan et al. designed a system composed of light‐emitting diodes, analog‐to‐digital circuits, and pressure sensors to achieve cross‐modal sensory.^[^ ^7^ ^]^ Wan et al. proposed a visual‐haptic perceptual system by combining a pressure sensor, a photodetector, and a synaptic transistor via ionic cable.^[^ ^8^ ^]^ The combination of different mechanical and optical sensors may require complex hardware circuitry and algorithms. Nonetheless, neuromorphic electronics can streamline the circuitry and even integrate diverse sensing functions (e.g., light, temperature, and pressure) in a single device, thus serving as the basis for the development of efficient artificial MSSs.^[^ ^11^ ^]^ Various types of neuromorphic electronic devices were developed to implement multimodal sensory functions, such as memtransistors, detectors, and memristors.^[^ ^11^, ^12^, ^13^ ^]^ For instance, Wang et al. developed a multimodal sensory organic electrochemical memtransistor by using mixed ionic‐electronic conductor components.^[^ ^11^ ^]^ In particular, Yang's group proposed an α‐In_2_Se_3_ optoelectronic memristor with controllable temporal dynamics and developed a multimode reservoir computing (MRC) system for neuromorphic multimodal sensory.^[^ ^12^ ^]^ Therefore, the exploration of MSSs' development in a single neuromorphic device represents cutting‐edge research.

Optoelectronic memristors provide a promising platform for application to neuromorphic MSSs because they possess the capability to process both electrical and optical information.^[^ ^12^, ^14^ ^]^ Various materials, including oxide, perovskites, and 2D materials, have been used to fabricate optoelectronic memristors.^[^ ^15^, ^16^ ^]^ Among them, 2D materials have been extensively studied owing to their advantages, such as high carrier mobility and broad spectral response.^[^ ^17^, ^18^, ^19^ ^]^ For example, Zhou et al. demonstrated a 2D h‐BN/WSe_2_ heterostructure‐based retinomorphic device with optical and electrical all‐in‐one perception for motion detection and recognition.^[^ ^19^ ^]^ 2D tellurene (Te) also offers excellent optical and electrical responses for developing optoelectronic detectors and field‐effect transistors.^[^ ^20^, ^21^, ^22^ ^]^ Recently, some advanced works reported Te‐based memristors that performed electrical resistive switching, which has drawn great interest for their potential memory applications.^[^ ^23^, ^24^ ^]^ However, the demonstration of 2D Te‐based optoelectronic memristors remains at an early stage.

Recent achievements indicate that the introduction of defects in material or floating gate structure can allow charge trapping and detrapping, thus achieving light‐controlled memristive behavior for 2D optoelectronic memristors.^[^ ^25^, ^26^ ^]^ The Solution plasma process (SPP) has been reported as a novel technology for surficial oxidation in various materials, in which the plasma in the solution can split water and generate uniform defects with high reducibility.^[^ ^27^, ^28^, ^29^, ^30^ ^]^ Therefore, the SPP method may offer a feasible way to introduce defects in 2D Te and fabricate an optoelectronic memristor. Benefiting from the controllable defect‐engineering strategy, the device can integrate both electrical and optical information within a single device, providing a reliable physical basis for the simulation of multimodal perception functions.

Herein, we demonstrated an SPP‐treated Te nanoflake‐based optoelectronic memristor, which can perform the short‐term synaptic plasticity under both the infrared (IR) optical and electrical stimulations. Furthermore, an MRC system with combined IR/electrical signals is constructed to perform visual and haptic multimode sensory and physiological monitoring tasks. The proposed Te nanoflake‐based optoelectronic memristive device provides an alternative candidate for developing more efficient neuromorphic MSSs.

## 2 Results and Discussion

### 2.1 SPP‐Treated Te Nanoflake‐based Optoelectronic Memristor

**Figure 1:**
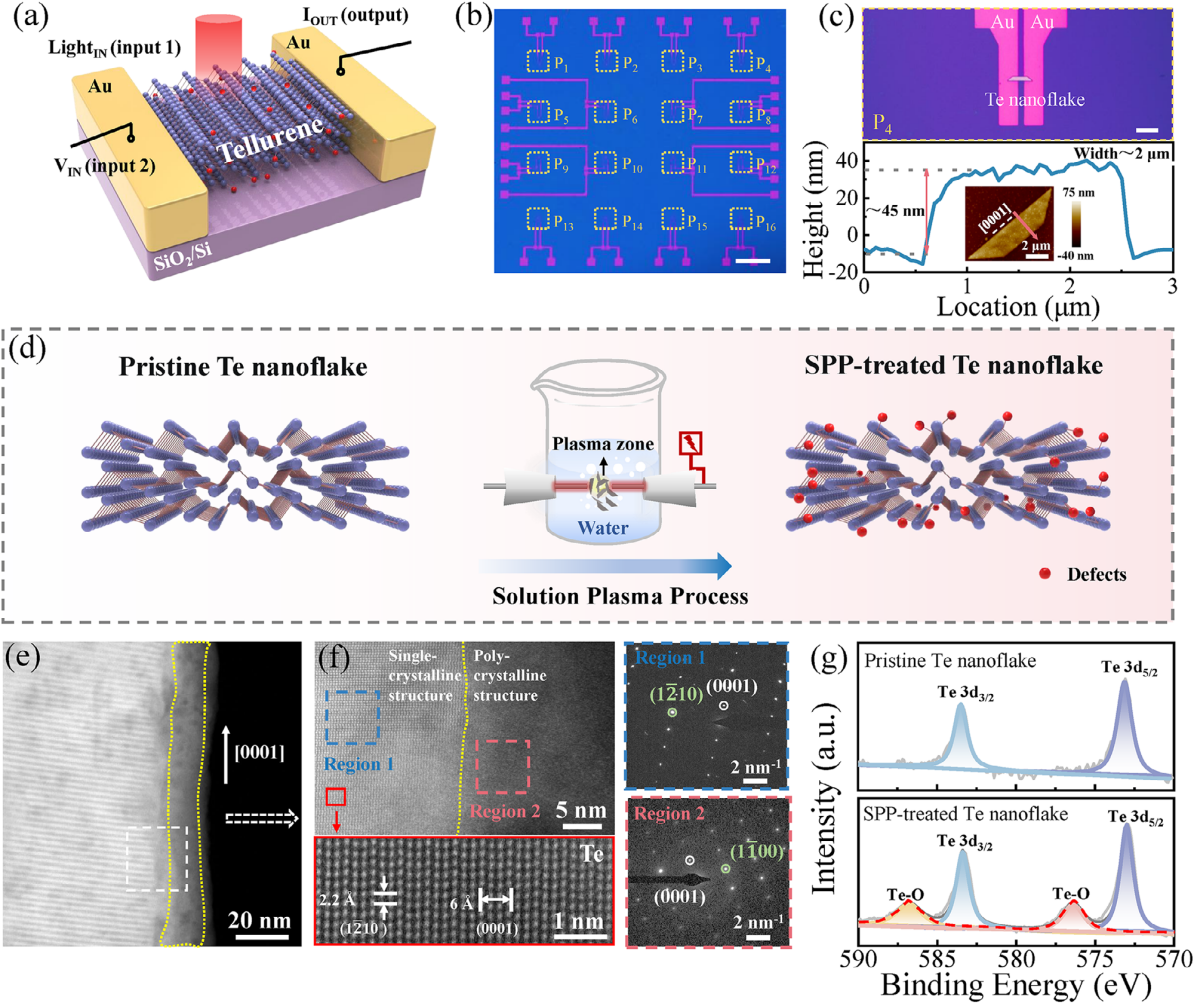
Schematic and characterization of the SPP‐treated Te‐based optoelectronic memristor. a) Structure of the SPP‐treated multilayer Te nanoflake‐based optoelectronic synapse. b) Optical image of the fabricated memristor array. Scale bar: 2 mm. c) Upper panel: the enlarged optical image of one single device. Scale bar: 10 µm. Bottom panel: AFM image and corresponding height profile of SPP‐treated Te nanoflake. d) Schematic of the SPP treatment in fabricating Te‐based optoelectronic memristor. e) HAADF‐STEM image of Te nanoflake after SPP treatment, and f) HAADF‐STEM image of the polycrystalline region with defects and the single‐crystalline region in the Te nanoflake. The right panels show the SAED patterns in the marked squares. g) The 3d core‐level XPS results of Te nanoflakes before and after SPP treatment.

**Figure** **1**a illustrates the SPP‐treated Te optoelectronic memristor with a two‐terminal Au/Te/Au planar structure in this study. Herein, the multilayer Te nanoflake was synthesized using a hydrothermal method and treated by SPP, then transferred onto a SiO_2_/Si substrate with patterned Au electrodes for device fabrication (see more details in Experimental Section). A 4 × 4 optoelectronic memristor array (marked as P1‐P16) was constructed as shown in Figure 1b, in which the distance between the paired Au electrodes is 3 µm for each single memristor device, as illustrated in the upper panel of Figure 1c. Figure S1 (Supporting Information) provides all the enlarged optical images of the memristor devices in Supporting Information. The thickness and width of Te nanoflakes are ≈45 and ≈2 µm, respectively, as characterized by the atomic force microscopy (AFM) image in Figure 1c and Figure S2 (Supporting Information). Importantly, the SPP method was adopted in the current work to introduce defects in Te nanoflakes for performing optoelectronic memristive behavior, as illustrated in Figure 1d. The plasma in the solution can split water and generate active radicals (e.g., hydroxyl, oxygen radical) with a certain oxidation potential, thereby creating surficial oxidation with defects in Te nanoflakes.^[^ ^29^, ^30^ ^]^ In addition, the defect concentration can be adjusted for device optimization by tuning the treatment time from 0.5 to 5 h. Therein, the preferred SPP treatment time is 2.5 h for the subsequent characterization and measurements.

In order to investigate the microscopic defects, the high‐angle annular dark‐field scanning transmission electron microscopy (HAADF‐STEM) image of SPP‐treated Te nanoflake is provided in Figure 1e. Pristine Te nanoflake generally has a uniform surface with a single‐crystalline structure (see Figure S3, Supporting Information). On the contrary, it can be seen clearly that the edge region (framed area in Figure 1e) with a width of ≈11 nm shows a different contrast from the central region in the SPP‐treated Te nanoflake. As shown in Figure 1f, for the central region, the HAADF‐STEM image and selected area electron diffraction (SAED) patterns indicate the single‐crystalline structure of Te nanoflake with the helical chains and a threefold screw symmetry along 〈0001〉. The interplanar spacings are 2.2 and 6.0 Å, corresponding to Te (1$\bar{2}$10) and (0001) planes, respectively.^[^ ^21^, ^31^, ^32^ ^]^ On the other hand, the edge region exhibits a polycrystalline structure with abundant defects, as confirmed by the HAADF‐STEM image and SAED pattern in the right panel of Figure 1f. A slight distortion appeared in the inverse Fourier‐filtered image with the use of SPP treatment, which results in the polycrystalline structure with a large number of defects in the Te nanoflakes (see Figure S4, Supporting Information). Furthermore, X‐ray photoelectron spectroscopy (XPS) analysis is conducted to study the defect chemistry of Te nanoflakes. As shown in Figure 1g, the intensity of Te 3d core‐level XPS spectra markedly changed after SPP treatment. In the Te nanoflake, two distinct peaks are typically observed at binding energies of 583.4 and 573.1 eV, which correspond to the Te 3d_3/2_ and Te 3d_5/2_ states, respectively.^[^ ^33^ ^]^ Notably, two more peaks at 586.5 and 576.1 eV are observed after SPP treatment, which are usually attributed to Te─O bonds in previous literature.^[^ ^33^, ^34^, ^35^ ^]^ This result is consistent with the reaction that occurred between Te and active radicals (e.g., hydroxyl radical, oxygen radical) in SPP. The above analysis indicates that the possible types of SPP‐induced defects are related to oxygen, such as substitutional defect (O_Te_) and interstitial oxygen (O_i_). These oxygen‐related defects play a great role in the optoelectronic memristive mechanism, which will be further discussed later.

### 2.2 Optoelectronic Synapse Performance and Mechanism

**Figure 2:**
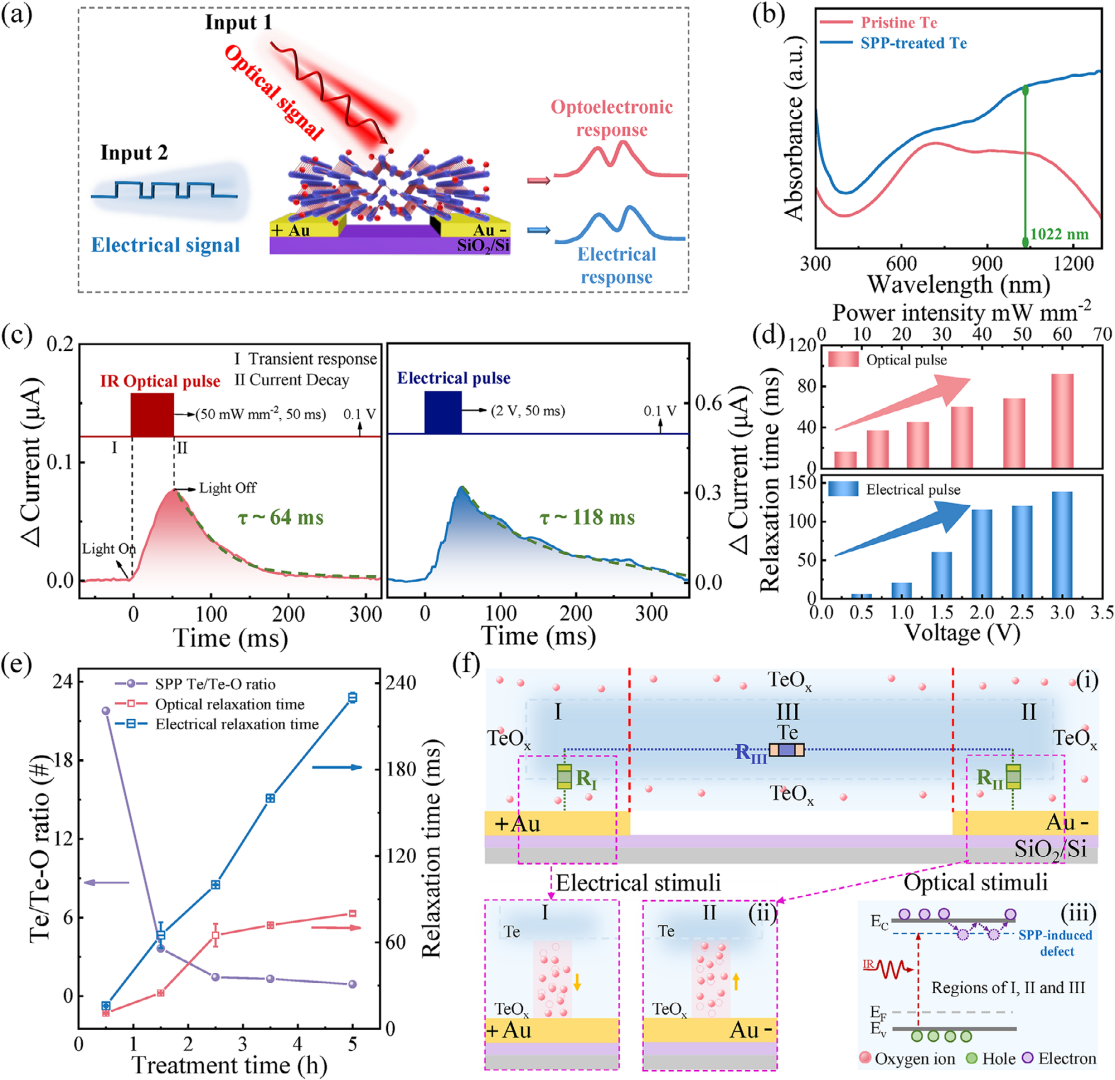
Device performance and switching mechanism of the SPP Te‐based optoelectronic synapse. a) Schematic of the optoelectronic memristor and experimental measurement set‐up. b) Absorption spectra of Te nanoflakes before and after SPP treatment. c) EPSCs are stimulated by a single optical pulse (50 mW mm^−2^, 50 ms) and a single electrical pulse (2 V, 50 ms). d) Dependence of relaxation time on the light illumination intensity and the electrical amplitude. e) Dependence of Te/Te‐O ratio and relaxation time on SPP treated time. f) Schematic diagram of the multimodal switching mechanism of the Te optoelectronic memristor.

To verify the optoelectronic synaptic performance, the transient optical and electrical measurements were conducted in this SPP‐treated Te memristor, as illustrated in **Figure** **2**a. Figure 2b shows the absorption spectra of the pristine Te and SPP‐treated Te nanoflakes. It can be seen that the absorption in the near‐infrared (NIR) range clearly increased after the SPP treatment, which could be explained by the introduction of oxygen‐related defects by SPP.^[^ ^28^, ^29^, ^30^ ^]^ The wavelength of 1022 nm is utilized in this study owing to the good response of 2D Te materials to IR light irradiation. As illustrated in Figure 2c, both the IR light pulse (50 mW mm^−2^, 50 ms) and electrical stimulations (2 V, 50 ms) can induce the transient increase of current (Δ*I*) similar to the excitatory postsynaptic current (EPSC) in neuroscience. The low fluctuation of EPSCs for the devices in the 4 × 4 memristor array can be achieved thanks to the uniformity of SPP‐treated Te nanoflakes (see Figure S5, Supporting Information). A spontaneous decay within tens of microseconds can be observed after the ΔI, indicating the emulation with short‐term plasticity (STP). Herein, the synaptic change ΔI in measurement is monitored by applying a bias voltage of 0.1 V. The relaxation time τ is extracted from the decay process by fitting the exponential function I = I_0_+I_A_exp(‐t/τ), where I_0_ and I_A_ are the offset current and fitting constant.^[^ ^36^, ^37^ ^]^ The relaxation times τ are 64 and 118 ms for single optical and electrical pulses, respectively. In fact, the relaxation time represents the critical temporal parameter to make the correlation between different spikes, thus allowing the building reservoir computing (RC) system. Figure 2d illustrates that both the optical‐induced and electrical‐induced relaxation time can be adjusted in the range of tens of microseconds, in which τ increases with the power density of light and the amplitude of the electrical pulse. Time variation curves of photocurrent were provided in Figure S6 (Supporting Information) under different light intensities and electrical amplitude. Importantly, the SPP‐treated Te optoelectronic synapse presents its potential to realize multimodal synaptic function under mixed optical and electrical stimulations based on the above results. The stimulation conditions of the IR light pulse (50 mW mm^−2^, 50 ms) and electrical pulse (2 V, 50 ms) are selected to operate the MRC later, which will be discussed in the next sections.

To investigate the multimodal switching mechanisms of the Te optoelectronic memristor, the dependence of relaxation time τ and Te/Te‐O ratio on SPP treatment time is studied, as shown in Figure 2e. It can be seen that both the optical and electrical relaxation time increased with increasing the SPP treatment time from 0.5 to 5 h, while the ratio of Te/Te‐O decreased from 21.78 to 0.9 (see Figure S7, Supporting Information). As discussed before, the oxygen‐related defects are generated by SPP, which is consistent with the decreased result of the Te/Te‐O ratio. Furthermore, it also indicates that the relaxation time τ has a close relationship with oxygen‐related defects. A possible mechanism model is proposed to explain the multimodal memristive switching for our Te optoelectronic memristor in the following. Figure 2f illustrates the Te optoelectronic memristor with the planar structure, in which the current transport can be ascribed to the contribution from three regions, as marked the regions I, II, and III. Considering the high conductivity of pristine single‐crystalline Te nanoflake, regions I and II can be regarded as two vertical devices with the structure of Te/TeO_x_/Au, while region III is regarded as one Te horizontal device with the top and bottom surfaces of Te/TeO_x_, as illustrated in Figure 2f‐i. Herein, the TeO_x_ means the Te layer with oxygen‐related defects, which has much higher resistance compared to the pristine Te (see Figure S8, Supporting Information). For regions I and II, they would play a great role in the electrical switching mode (Figure 2f‐ii), where the electrical field of the pulse drives the migration of oxygen‐related defects, thus decreasing the defect concentration and the resistances of R_I_ and R_II_. Regions abundant in oxygen vacancies develop high‐conductance pathways, while the other areas stay at low conductance.^[^ ^38^ ^]^ Additionally, the current decay after withdrawing the electrical pulse can be explained by the diffusion of these migrated defects. In fact, the above model for electrical resistive switching is consistent with the classical conducting filament model, as addressed in the literature.^[^ ^38^, ^39^ ^]^ These internal defect dynamics enable the memristor to demonstrate several key short‐term synaptic behaviors, as discussed below. On the other hand, the optical switching mechanism in our device may be attributed to the charge trapping/detrapping by the oxygen‐related defects in the regions of I, II, and III, as illustrated in Figure 2f‐iii. As optical stimulations are applied, the electrons are excited from the valence band to the conduction band of the Te nanoflake, thus increasing the transient hole density and device current corresponding to EPSC. Importantly, these excited electrons could be trapped by oxygen‐related defects, which hinder the recombination of electrons and holes, producing persistent photoconductivity. Subsequently, the current decay occurs by detrapping these electrons from defects. Similar charge trapping/detrapping mechanisms for optoelectronic memristors were also widely reported in previous works.^[^ ^40^, ^41^ ^]^ From the above results, the SPP‐treated Te optoelectronic memristor demonstrates both the electrical and optical synaptic plasticity with temporal dynamics, which offers a platform to process multimodal information.

### 2.3 Temporal Information‐Linked Optical and Electrical Signals

**Figure 3:**
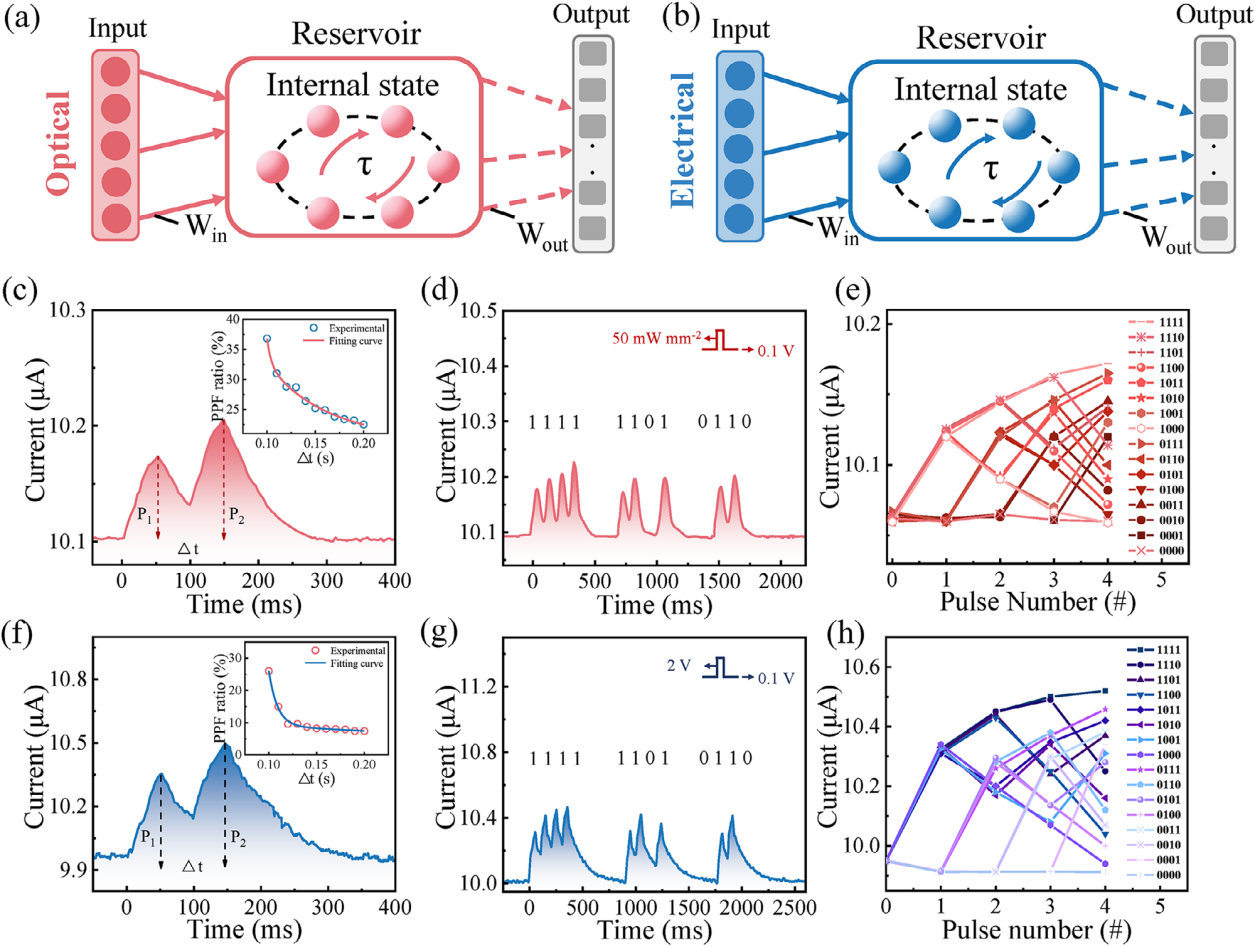
Single‐modal RC systems with optical and electrical inputs. a,b) Schematics of the optical‐input and electrical‐input RC systems. c) PPF induced by IR optical stimulations (1022 nm, 50 mW mm^−2^, 50 ms). Inset: PPF index as a function of the time interval Δt. d) Current responses of our device under optical stimuli to three typical pulse streams, including [1111], [1101], and [0110]. e) Summary of the device responses to all 4‐bit patterns under optical stimuli. f) PPF induced by electrical paired pulses (2 V, 50 ms) and its index as a function of the time interval Δ*t*. g,h) Device responses to the electrical patterns of [1111], [1101], and [0110], and the summary of device responses to all the patterning streams under electrical stimulations.

RC system is a highly efficient network for processing temporal tasks, such as speech/visual/motion recognition, time series prediction and others.^[^ ^42^, ^43^ ^]^ Before implementing the MRC system for neuromorphic multimodal perception, the single‐mode RC system with only optical or electrical input is studied using the SPP‐treated Te optoelectronic memristor, as presented in **Figure** **3**a,b. According to the result of Figure 2c, the Te memristor is able to process temporal information under optical and electrical signals. In order to evaluate the dynamic range that can make the temporal correlation between two individual stimulations, the classical paired‐pulse facilitation (PPF) function is implemented by using a pair of optical pulses (50 mW mm^−2^, width of 50 ms) with different intervals (Δ*t*), as illustrated in Figure 3c. For the PPF function, the second spike can induce a higher peak value (P_2_) than the first one (P_1_), indicating the existence of temporal influence from the first spike. As shown in the inset of Figure 3c, the higher PPF ratio (PPF ratio = (P_2_‐P_1_)/P_1_×100%) can be induced by the shorter time interval Δt, whose correlation can be well fitted with an exponential decay equation similar to that of a biological synapse.^[^ ^41^, ^44^ ^]^ To further present the device's potential application for the RC system, a 4‐bit binary stream with different encoding patterns was applied on the device using optical stimuli, as illustrated in Figure 3d,e. The corresponding current responses were recorded using a source meter. Figure 3d shows the device response under stimulations of [1111], [1101], and [0110], in which the current response of these three typical patterns can be clearly distinguished, presenting its capability for processing sequential data. Furthermore, a pivotal attribute of the RC system is the presence of abundant reservoir states, which are typically essential for influencing the system's overall performance. To validate the presence of reservoir states within the device, Figure 3e presents the distinct evolutionary trajectories corresponding to different 4‐bit patterns, which underscores the device's exceptional ability to differentiate among 16 distinct reservoir states for temporal recognition. Similarly, the Te memristor is adept at executing the PPF function and recognizing 4‐bit patterns through electrical stimulation (2 V, 50 ms), as depicted in Figure 3f–h. The uniformity experiments of the optoelectronic memristor were provided in Figures S9–S11 (Supporting Information). Significantly, the ability to perform RC with both electrical and optical signals opens up new avenues for developing an MRC system that can handle mixed input signals, leveraging our memristor technology.

### 2.4 MRC System for Digital Pattern Recognition

**Figure 4:**
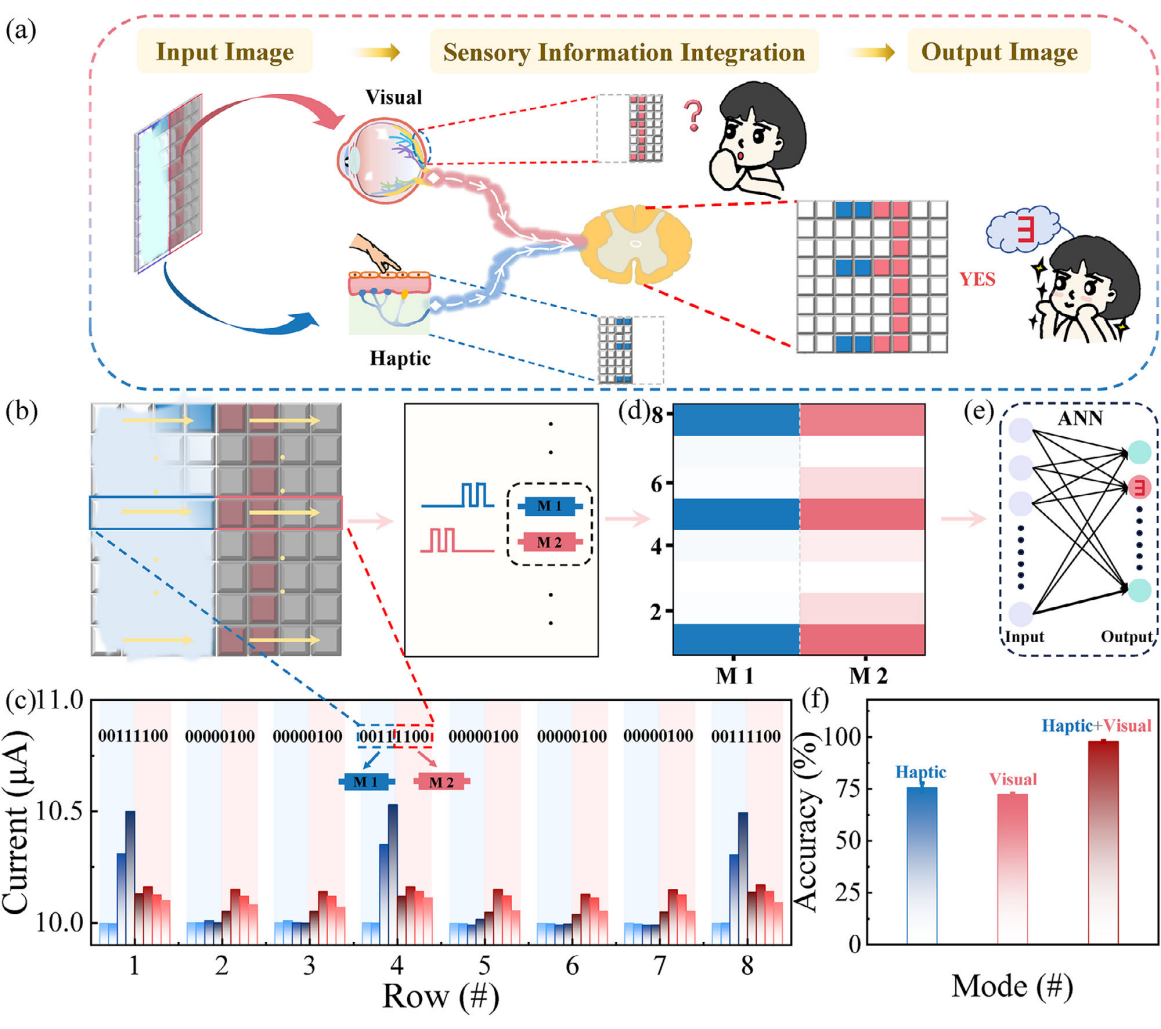
Visual‐haptic multimodal perception implemented by the MRC system. a) Schematic of the multimodal perception using the MRC system. b) The digital pattern contains mixed visual‐haptic inputs. The left portion of the pattern is covered in paint and can be sensed only by haptic information, while the right half is perceived through visual sensing. The haptic and visual sensing correspond to electrical and optical signals. c) The current responses of MRC to the digital sequences derived from (b). d) The mapping of current response during the stimulation of mixed inputs. e) The current values collected after each electrical and optical sequence are fed into an ANN for classification. f) The recognition accuracy of MRC for different modes: haptic, visual, and visual‐haptic modes.

Neuromorphic multimodal perception endows biological sensory systems with heightened sensitivity, enabling them to operate effectively in complex environments, e.g., visual‐haptic perception, as illustrated in **Figure** **4**a. To validate the multimodal perception capabilities of our memristor, the Te optoelectronic memristor‐based MRC system is examined for its ability to execute digital pattern recognition using visual‐haptic inputs, as demonstrated in Figure 4b–e. Figure 4b displays the digital input pattern incorporating mixed visual‐haptic inputs, with the left half corresponding to only electrical signals and the right half to optical signals. In the experimental setup, a piezoelectric nanogenerator was employed to transform haptic information into electrical pulses (see Figure S12, Supporting Information). As shown in Figure 4b,c, the input pattern (8×8 pixels) was organized into 8 rows, with each row comprising two pulse sequences – each consisting of four electrical pulses and four optical pulses‐applied on two separate memristors for MRC. The 16 devices of our memristor array are utilized to implement this MRC.

Using the digital pattern “3” and row 4 as an example, the sequences “0011” and “1100” are respectively applied to the two memristors via electrical and optical pulses. Figure 4c also presents the complete current responses of the memristors within the MRC system in response to the applied mixed‐input sequences. Figure 4d summarizes the final reservoir state of each sequence into a current mapping for subsequent pattern classification, in which column M1 denotes the final current values associated with the electrical response and column M2 denotes those related to the optical response (Figure 4e). The training data and testing data were conducted on 2000 and 500 samples. The training and test datasets were constructed by applying randomly generated salt‐and‐pepper noise to the original images. Herein, the final current values obtained after each sequence are input into an artificial neural network (ANN) for the purpose of pattern classification (see Figure S13, Supporting Information). Figure 4f offers a comparative analysis of recognition accuracy across various modes, encompassing haptic, visual, and visual‐haptic modes. It is evident that the haptic and visual modes can only obtain an accuracy of 75.6% and 72.4%, respectively, as they rely on only partial information. In contrast, the visual‐haptic modes, which combine both the visual and haptic data, significantly enhance the accuracy to 97.8%. The above result demonstrates that the Te optoelectronic memristor‐based MRC system is adept at facilitating neuromorphic multimodal perception, leading to highly accurate recognition.

**Figure 5:**
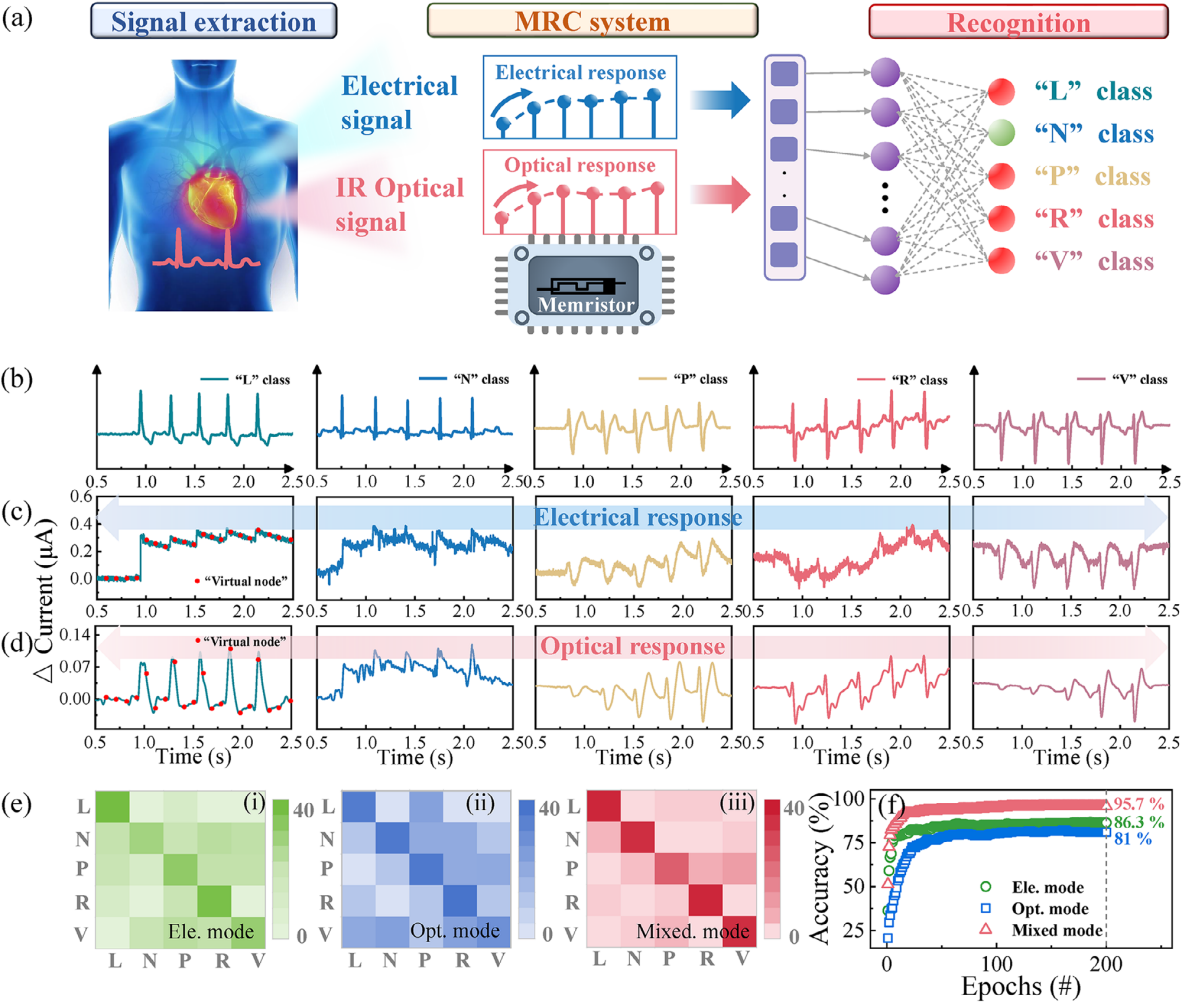
ECG pattern recognition using the MRC system. a) Schematic of the MRC system for ECG pattern recognition. It contains the signal extraction, reservoir functionality, and the readout neural network. b) Five classes of ECG waveforms with distinct temporal information, including “L,” “N,” “P,” “R,” and “V.” c,d) The current response of MRC to the electrical (2 V, 50 ms) and optical stimuli (50 mW mm^−2^, 50 ms) with features of five ECG patterns. e) Confusion matrices and f) recognition accuracy of ECG patterns in electrical‐input, optical‐input, and mixed input modes.

The ability to process the temporal multimodal data is an essential feature of our Te optoelectronic memristor‐based MRC system, which could potentially facilitate applications in physiological signals monitoring, such as electrocardiogram (ECG) analysis. ECG is a diagnostic technique that captures the electrical activity of the heart, providing insights into the functionality of the cardiovascular system.^[^ ^45^, ^46^, ^47^, ^48^, ^49^ ^]^ Achieving high recognition accuracy for ECG patterns is essential due to the subtle differences present in arrhythmia signals, and our MRC system may offer a feasible solution to meet this requirement. **Figure** **5**a provides a schematic representation of the Te optoelectronic memristor‐based MRC system for ECG pattern recognition. This system typically includes three stages: signal extraction, reservoir, and recognition. As a proof of concept, five typical classes of ECG patterns were extracted from a heartbeat MIT‐BIH dataset for subsequent recognition, including “left bundle branch block beat (L)”, “normal (N)”, “paced beat (P)”, “right bundle branch block beat (R)”, and “premature ventricular contraction (V)”.^[^ ^46^ ^]^ These ECG patterns were initially preprocessed to achieve a peak‐to‐peak period of ≈350 ms, which corresponds to the temporal characteristics of our MRC system, as shown in Figure 5b. Subsequently, they were fed into an arbitrary waveform generator and a laser to produce electrical and IR optical pulses. Figure 5c,d depicts the current response of the MRC system to electrical and optical pulses, showcasing the real‐time variation of current in response to ECG signals. This pattern, which consists of temporal dynamic data, differs from the digital pattern results shown in Figure 4c. Consequently, prior to conducting ECG pattern classification, the continuous data must be transformed into virtual modes, as referenced in previous literature^[^ ^50^ ^]^ and indicated in Figure 5c,d. For example, an ECG pattern spanning 2 s was segmented into twenty virtual node states (red dots), with a 100 ms interval separating each pair of virtual nodes. Furthermore, these patterns with virtual nodes are introduced into an ANN acting as the readout layer with five output neurons (sigmoid activation function) for pattern classification using the back‐propagation algorithm (see Figure S14, Supporting Information). An activated output neuron serves as the final processing unit that produces the output for a given input. Figure 5e,f displays the confusion matrices and recognition accuracy of the ECG pattern under electrical‐input, optical‐input, and mixed‐input modes. It is evident that the MRC system clearly enhances the recognition accuracy to 95.7% after 200 training sessions through multimodal processing, surpassing the initial accuracy rates of 86.3% and 81% achieved with single‐modal approaches. These results indicate that the Te optoelectronic memristor‐based MRC system holds promise as a high‐precision multimodal sensory platform, offering valuable insights for healthcare applications.

## 3 Conclusion

In conclusion, we have demonstrated an SPP‐treated Te‐based optoelectronic memristor for emulating multimodal synaptic functions and have developed an associated MRC system. This device can replicate various short‐term plasticities, such as STP, EPSC, and PPF, using both optical and electrical stimuli. The MRC system is adept at processing multimodal signals, including the integration of visual and haptic sensory inputs, as well as ECG pattern classification. The experimental data reveal that the MRC system's recognition accuracy relying on multimodal perception can reach up to 95.7% for ECG pattern classification. Our research presents a viable approach to fabricating 2D Te‐based optoelectronic memristors through SPP treatment, which is pivotal for the advancement of high‐precision neuromorphic multimodal perception systems.

## 4 Experimental Section

### Synthesis of 2D Te Material

The 2D Te nanoflakes were synthesized following the method reported in the literature.^[^ ^21^ ^]^ First, the homogeneous solution was prepared by containing Na_2_TeO_3_ (0.05 g), polyvinyl pyrrolidone (PVP), and double‐distilled water. Second, an ammonia solution (4 mL) and hydrazine hydrate (1 mL) were added to the solution. Then, the mixture was poured into a 100 mL Teflon‐lined stainless‐steel autoclave and maintained at a temperature of 240 °C for 36 h, and 2D Te nanoflakes were eventually obtained.

### SPP Treatment

The synthesized Te nanoflakes were placed into a double‐layered quartz reactor with 100 mL of deionized water. The electrodes consisted of tungsten rods and tubes with an outer diameter of 3 mm and an inner diameter of 1 mm (purity 99%), where the distance between the tips of the two electrodes was 0.2 mm. The SPP‐treated samples were collected through centrifugation and lyophilization.

### Device Fabrication

An optoelectronic memristor device with the structure of Au/Te/Au was fabricated on a SiO_2_/Si substrate. First, patterned Au electrodes were prepared with a distance of 3 µm between a pair of electrodes using photolithography and thermal evaporation. Secondly, thin Te nanoflakes were extracted from a dispersion with SPP‐treated Te nanoflakes and placed onto a polydimethylsiloxane (PDMS) layer. Then, the Te nanoflakes were transferred onto patterned Au electrodes by heating PDMS to 353 K, thus fabricating the optoelectronic memristor device.

### Experimental Measurements

The thicknesses of the SPP Te nanoflakes were characterized via AFM (Dimension Icon, Bruker). The optical absorbance properties of the pristine and SPP‐treated Te samples were recorded using a Jasco UH‐4150 spectrophotometer operating in integrating sphere mode. The electrical and optoelectrical measurements were conducted by an integrated testing platform containing a source meter (Keithley 2636b), a TGA12104 arbitrary waveform generator, a Tektronix DPO 70404C digital oscilloscope, a laser (1022 nm), a microscope, and others. All electrical measurements were performed at room temperature (24 °C) and 30% relative humidity in this work.

## Conflict of Interest

The authors declare no conflict of interest.

## Supporting information



Supporting Information

## Data Availability

The data that support the findings of this study are available from the corresponding author upon reasonable request.
