# Supplementary material for: 2D Tellurene‐Based Optoelectronic Memristor with Temporal Dynamics for Multimodal Reservoir Computing System
Source: Adv Sci (Weinh). 2025 Sep 4;12(43):e13647. doi: 10.1002/advs.202513647 (PMC12631935; doi:10.1002/advs.202513647)
Supplement: Supplementary file 1 — Supporting Information [file ADVS-12-e13647-s001.docx]

Supporting Information

**Title: 2D Tellurene‐Based Optoelectronic Memristor with Temporal Dynamics for Multimodal Reservoir Computing System**

Jingyao Bian^1,2^, Zhuangzhuang Li^1^, Shuang Liang^1^, Ye Tao^1,*^, Zhongqiang Wang^1,*^, Yongxing Zhu^1^, Changhua Wang^1^, Haiyang Xu^1,*^ and Yichun Liu^1^

^1^State Key Laboratory of Integrated Optoelectronics, Key Laboratory of UV Light-Emitting Materials and Technology of Ministry of Education, Northeast Normal University, Changchun, Jilin 130024, China

^2^School of Science, Heilongjiang University of Science and Technology, Harbin 150020, China

E-mail: [taoy506@nenu.edu.cn](mailto:taoy506@nenu.edu.cn); [wangzq752@nenu.edu.cn](mailto:wangzq752@nenu.edu.cn); [hyxu@nenu.edu.cn](mailto:hyxu@nenu.edu.cn);

Keywords: 2D materials, neuromorphic perception system, optoelectronic memristor, solution plasma process, tellurene


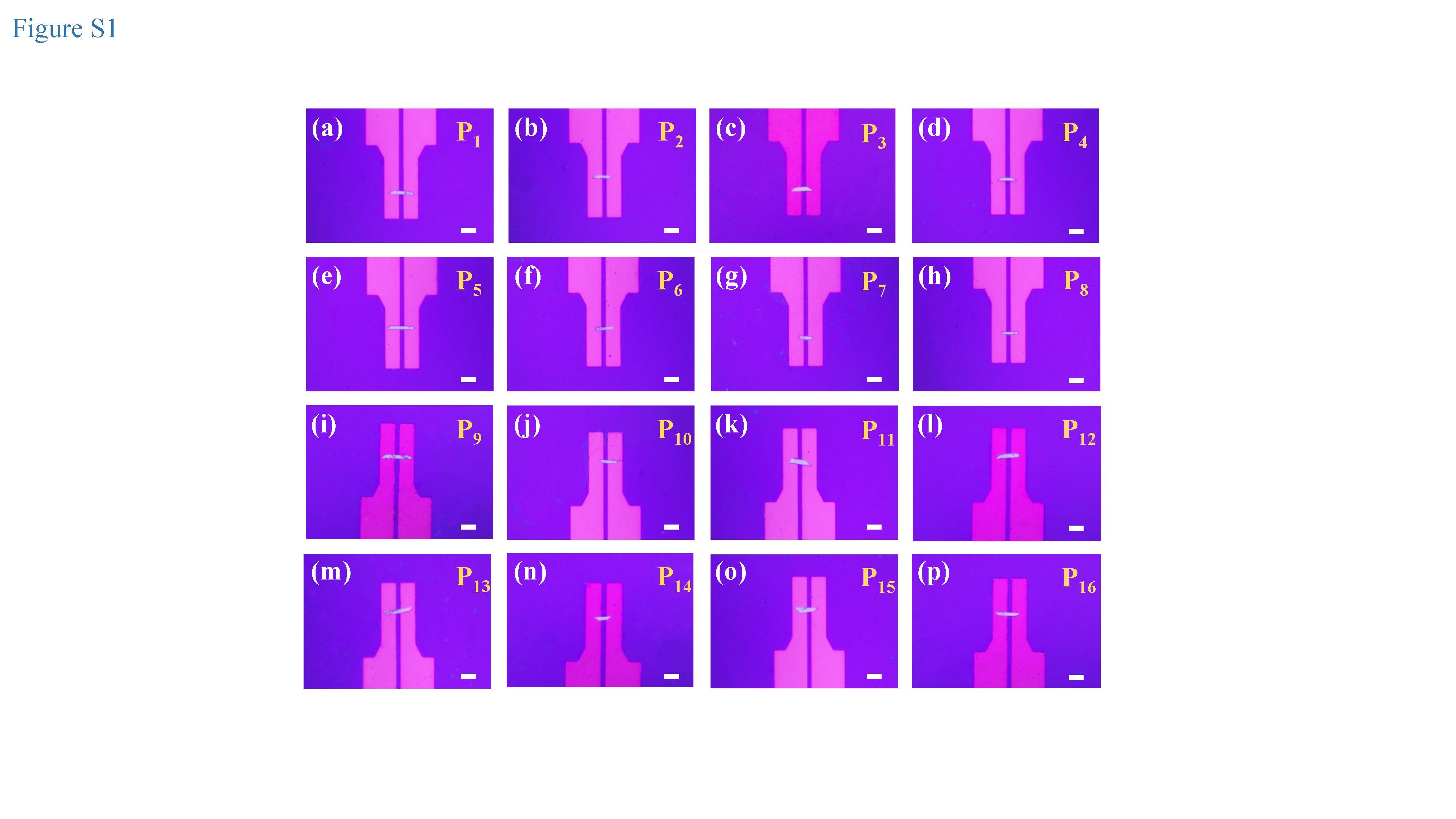


Figure S1. (a-p) The enlarged optical images of 16 devices in the memristors array, Scale bar: 10 μm.


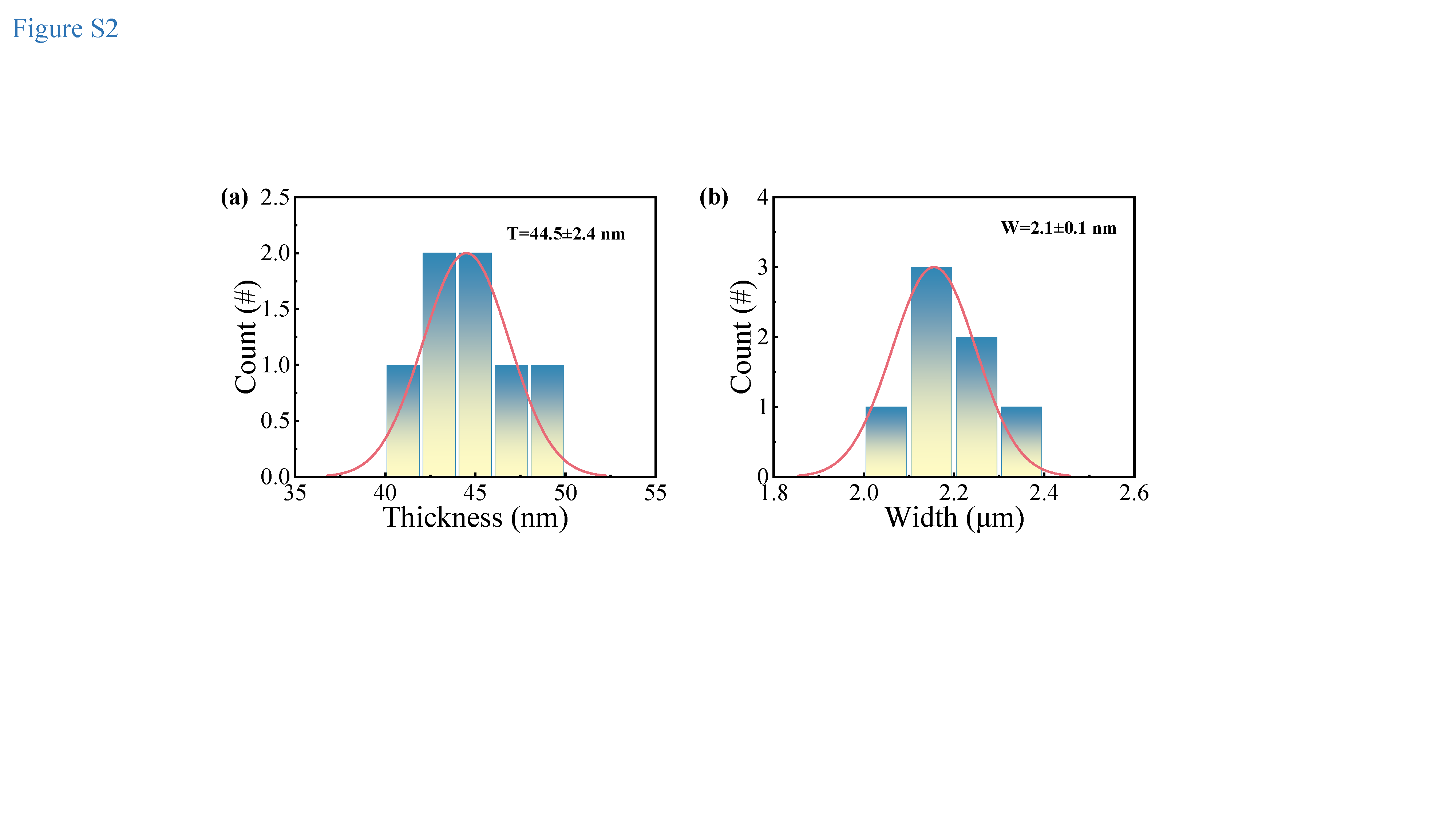


Figure S2. (a) and (b) The statistical data of the thickness and width for the SPP-treated Te nanoflakes show that the average thickness and width are 44.5 nm and 2.1 μm, respectively.


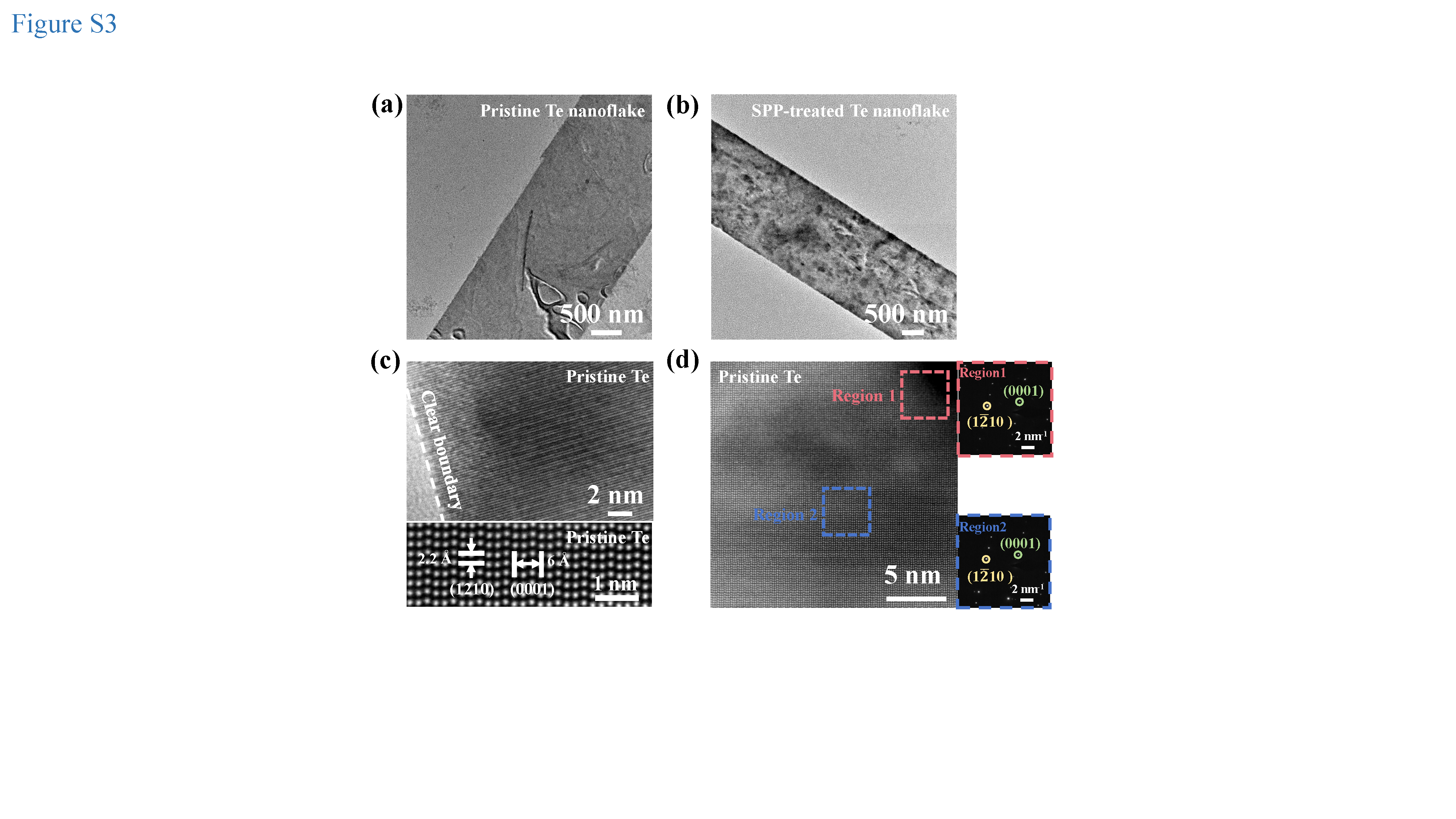


Figure S3. (a) and (b) TEM morphology images of the Te nanoflake before and after SPP treatment. (c) HRTEM image of the pristine Te nanoflake. (d) HAAD-STEM image of the single-crystalline structure of the pristine Te nanoflake. The right panels show the SAED patterns in the marked squares.

Figures 3(a) and (b) show the high-resolution transmission electron microscope (HRTEM) morphology image of the 2D Te nanoflake before and after SPP treatment. Figure 3(c) illustrates that the pristine Te nanoflake presents the typical single-crystalline structure. As shown in Figure 3(d), the HAADF-STEM image and SAED patterns indicate the single-crystalline structure of Te nanoflake with interplanar spacings are 2.2 Å and 6.0 Å, corresponding to Te (1$\bar{2}$10) and (0001) planes, respectively.


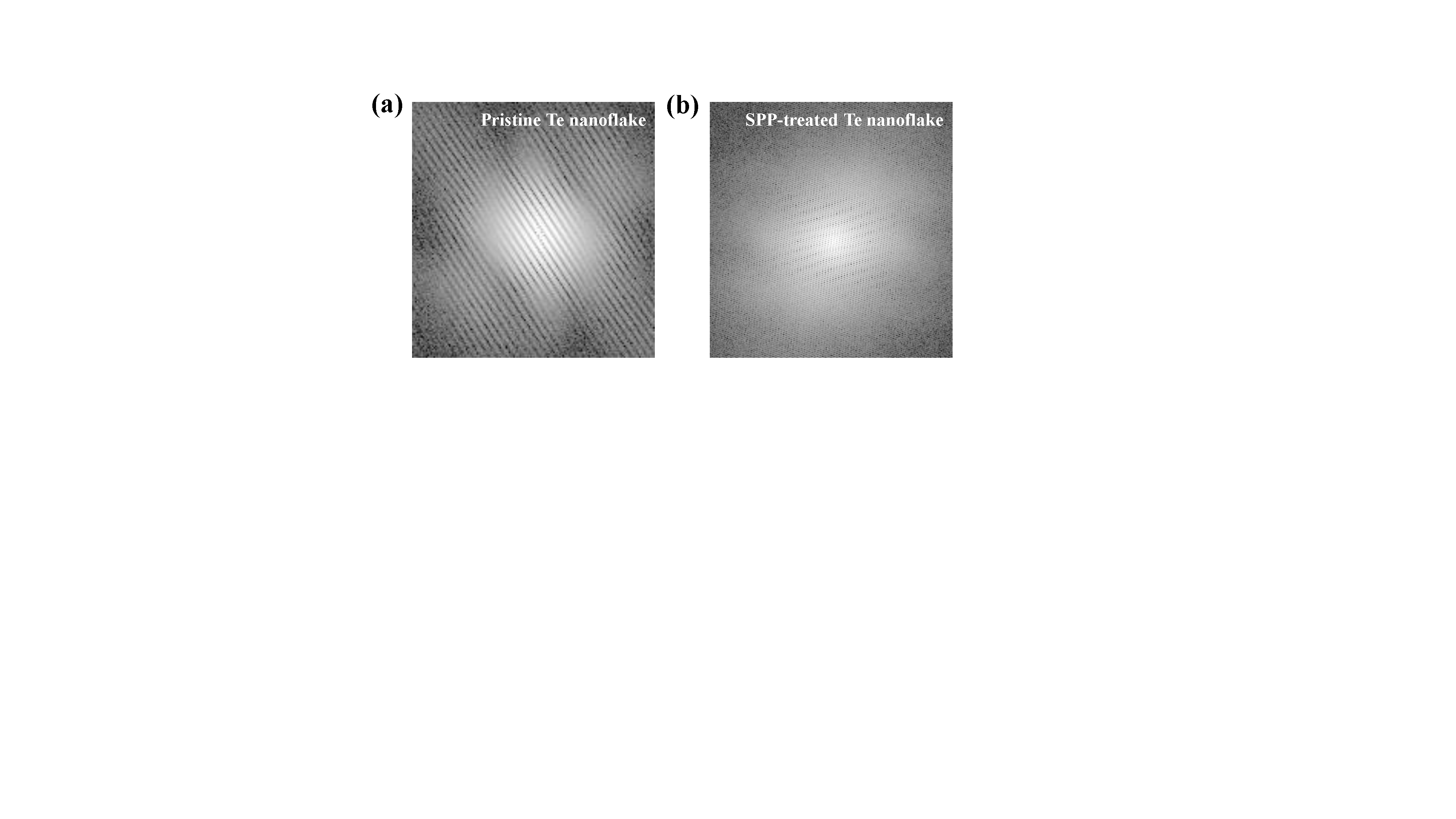


Figure S4. (a) and (b) The Fourier-filtered image (inverse FFT) of the (0001) regions marked in Figure 1 (f).


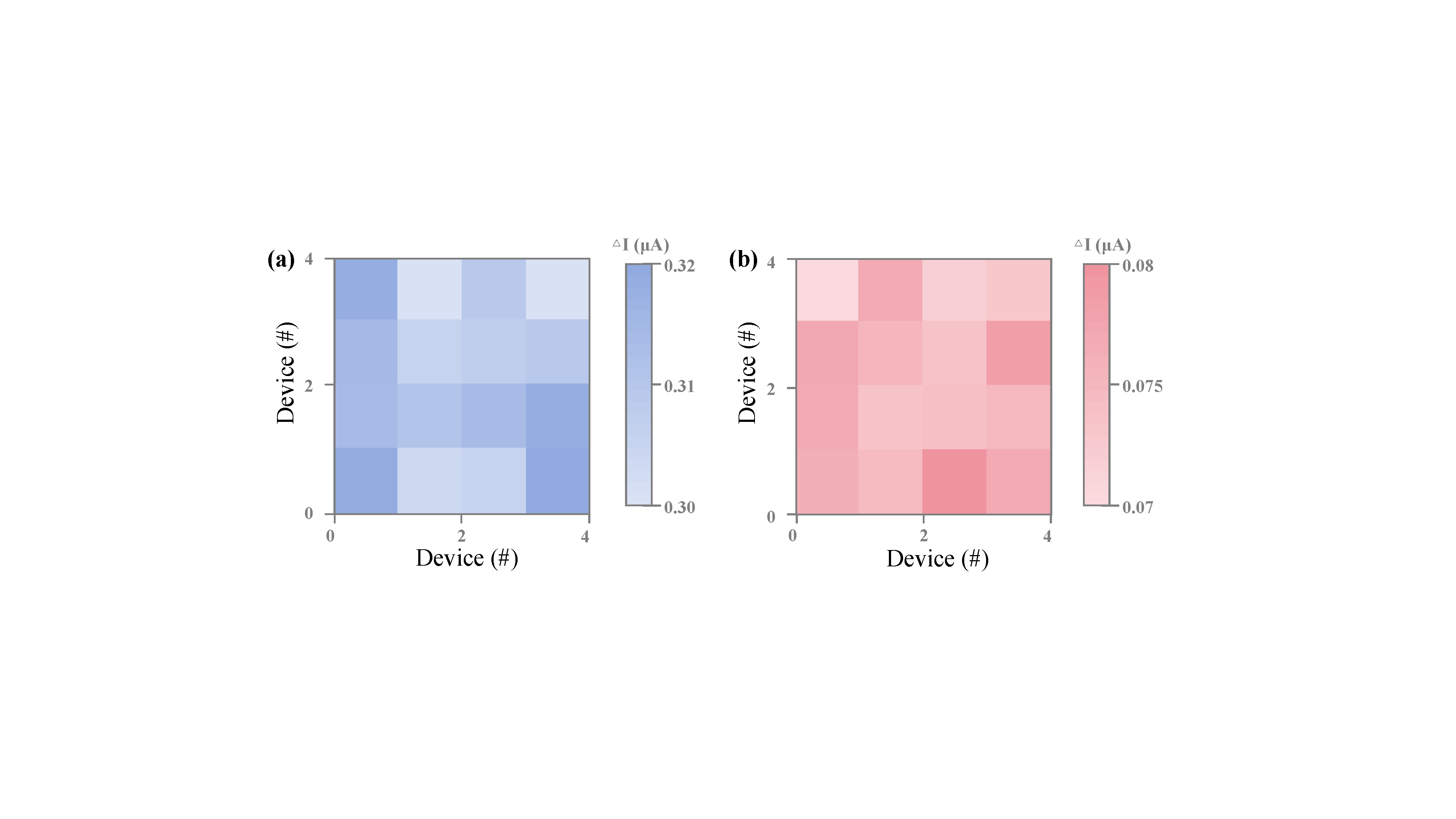


Figure S5. (a) and (b) Color mapping of EPSC values induced by electrical and optical stimuli by collecting data from 16 memristor devices.

To present the device-to-device variability, Figures 5(a) and (b) show the statistical ΔI results of EPSC induced by electrical and optical by collecting data from 16 memristor devices. The low current fluctuations 2.3% (σ/μ) and 4.1% (σ/μ) can be realized thanks to the good uniformity of the SPP-treated Te nanoflakes.


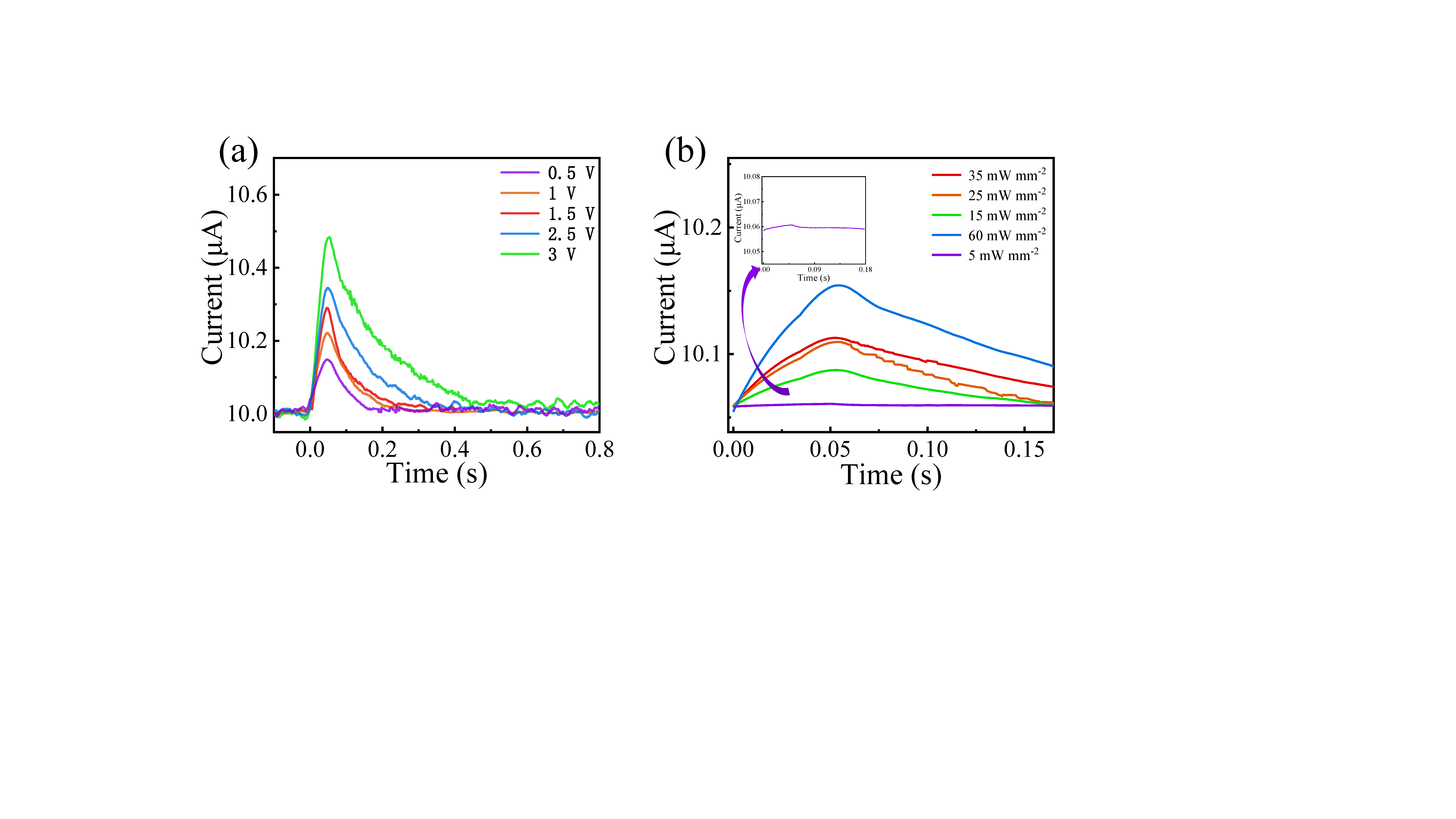


Figure S6 The dependence of relaxation time on light illumination intensity and electrical amplitude.


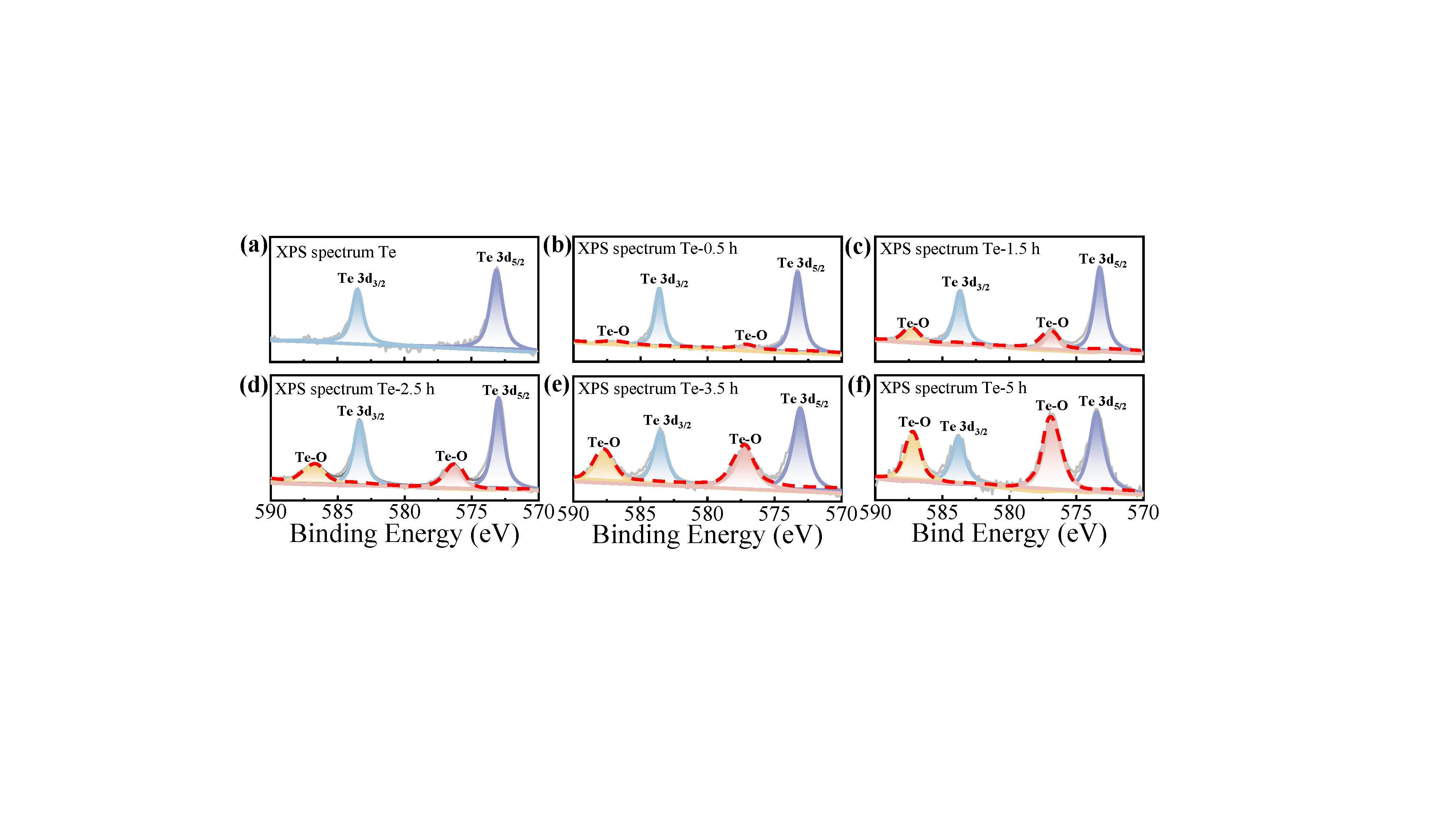


Figure S7. (a-f) Te 3d core-level XPS results of the SPP-treated Te nanoflake with different treatment times from 0 h to 5 h.


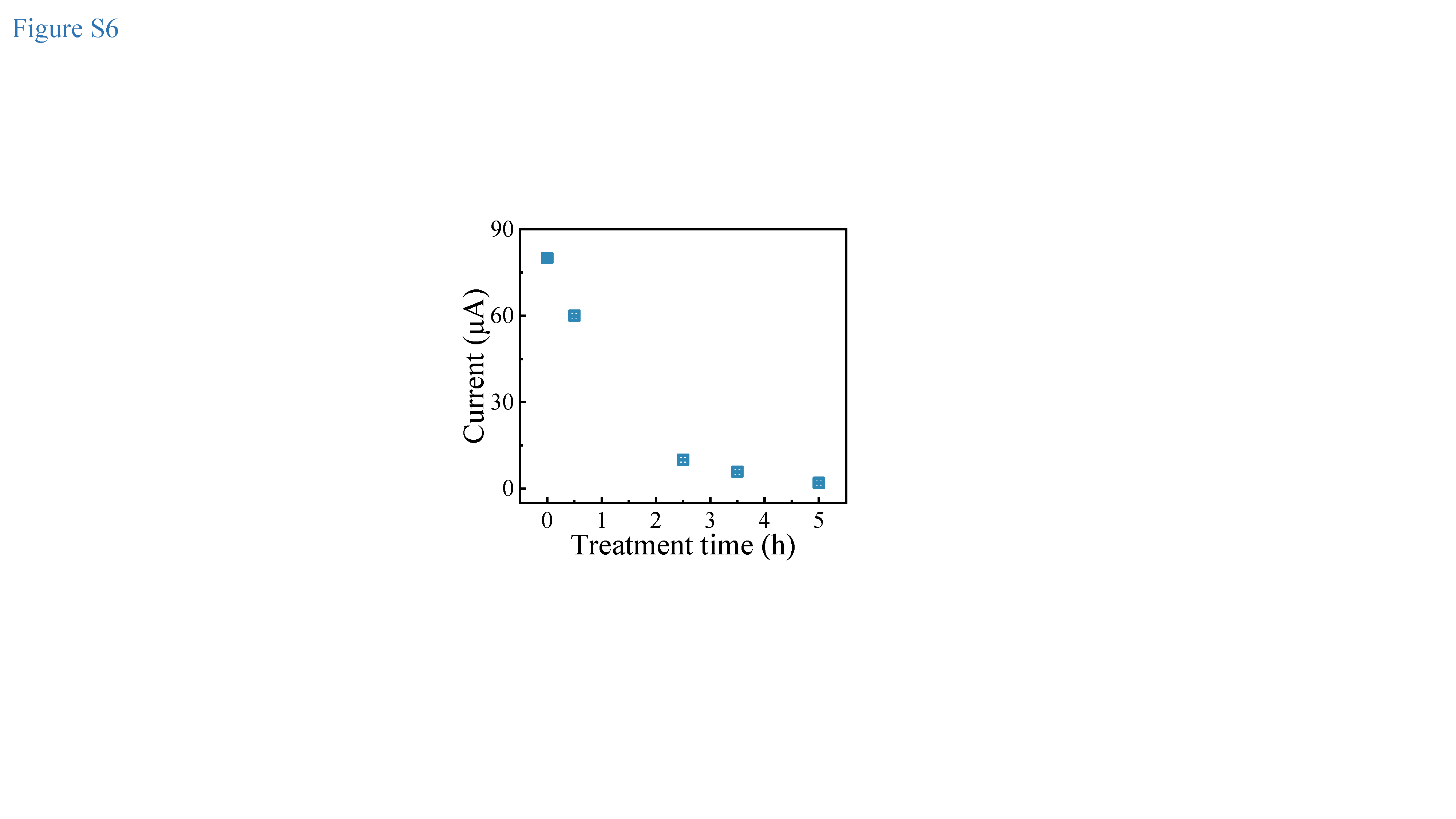


Figure S8. Dependence of the initial resistance of the SPP-treated Te memristive devices on SPP treatment time.

In order to confirm the influence of oxygen-related defects, we fabricated a series of SPP-treated Te memristive devices with different treatment times. As shown in Figure S8, the initial resistance of the SPP-treated Te memristive devices increases with increasing the SPP treatment time from 0 h to 5 h, which may be attributed to the introduction of oxygen-related defects by SPP.


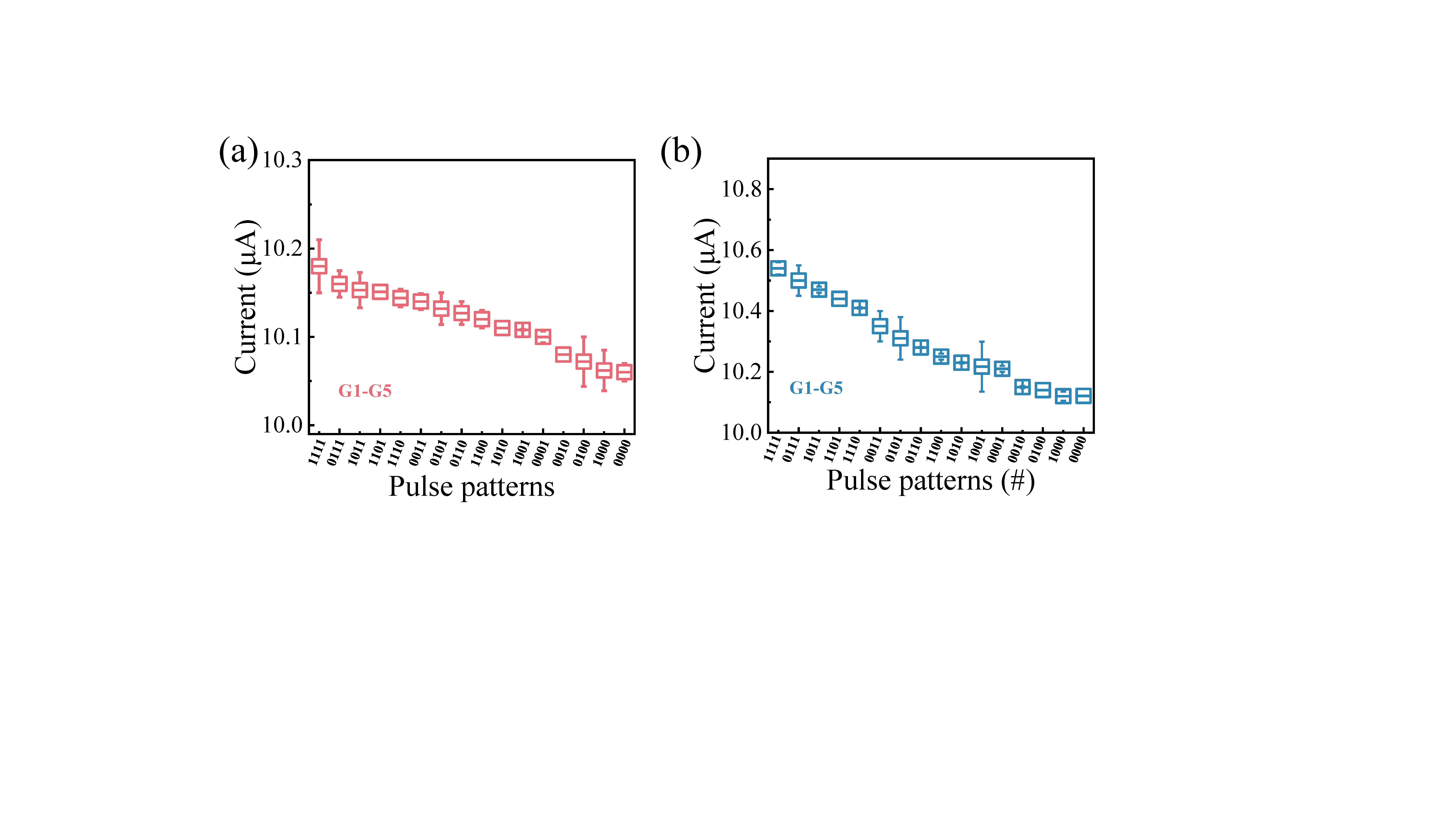


Figure S9 (a) and (b) Plots of the device current with the different pulse patterns in averaged over five devices (G1–G5) under optical pulse and electrical pulse.


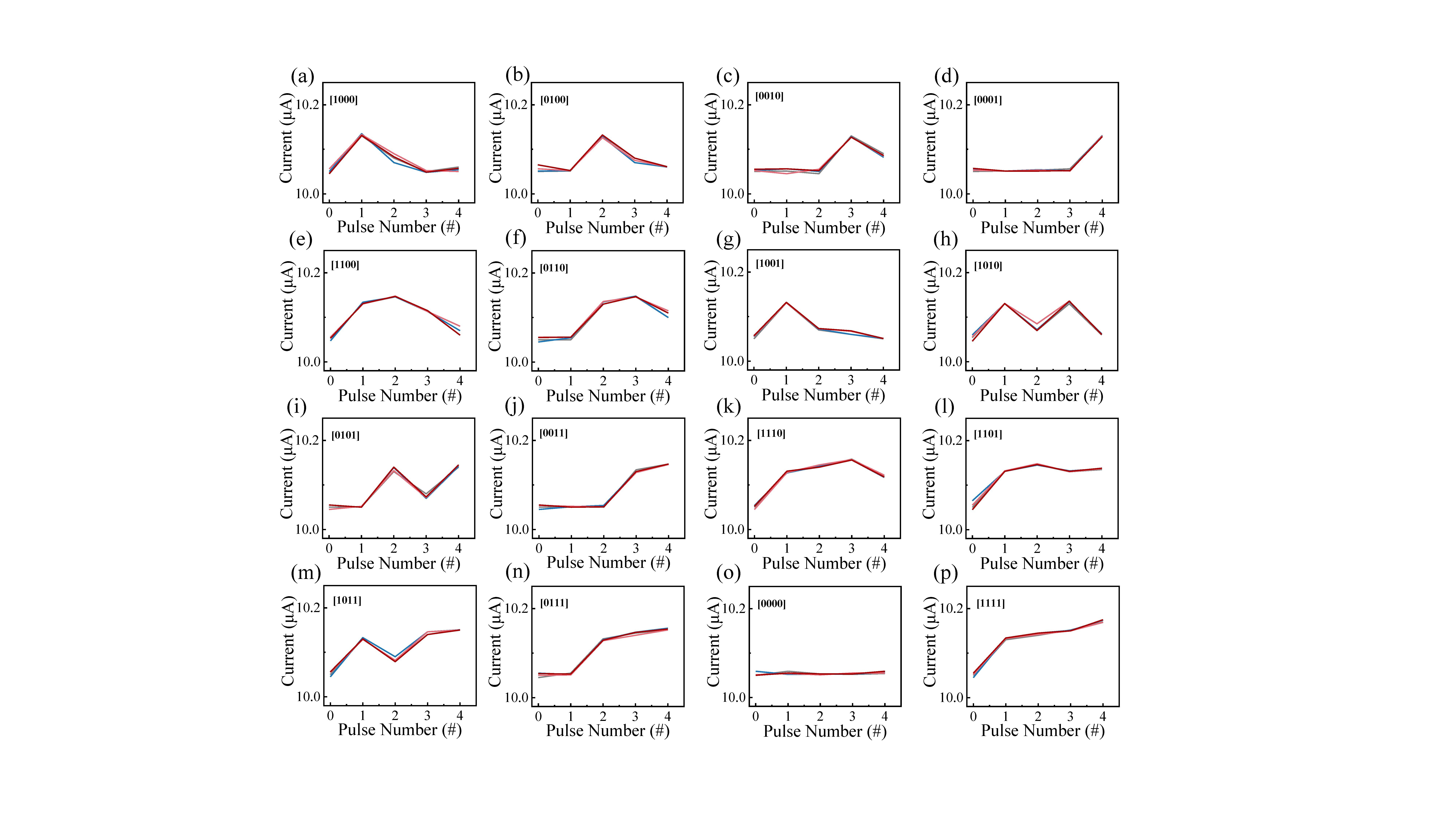


Figure S10 (a)-(p) Experimental read-current responses of Te-based optoelectronic memristor by optical input signals.


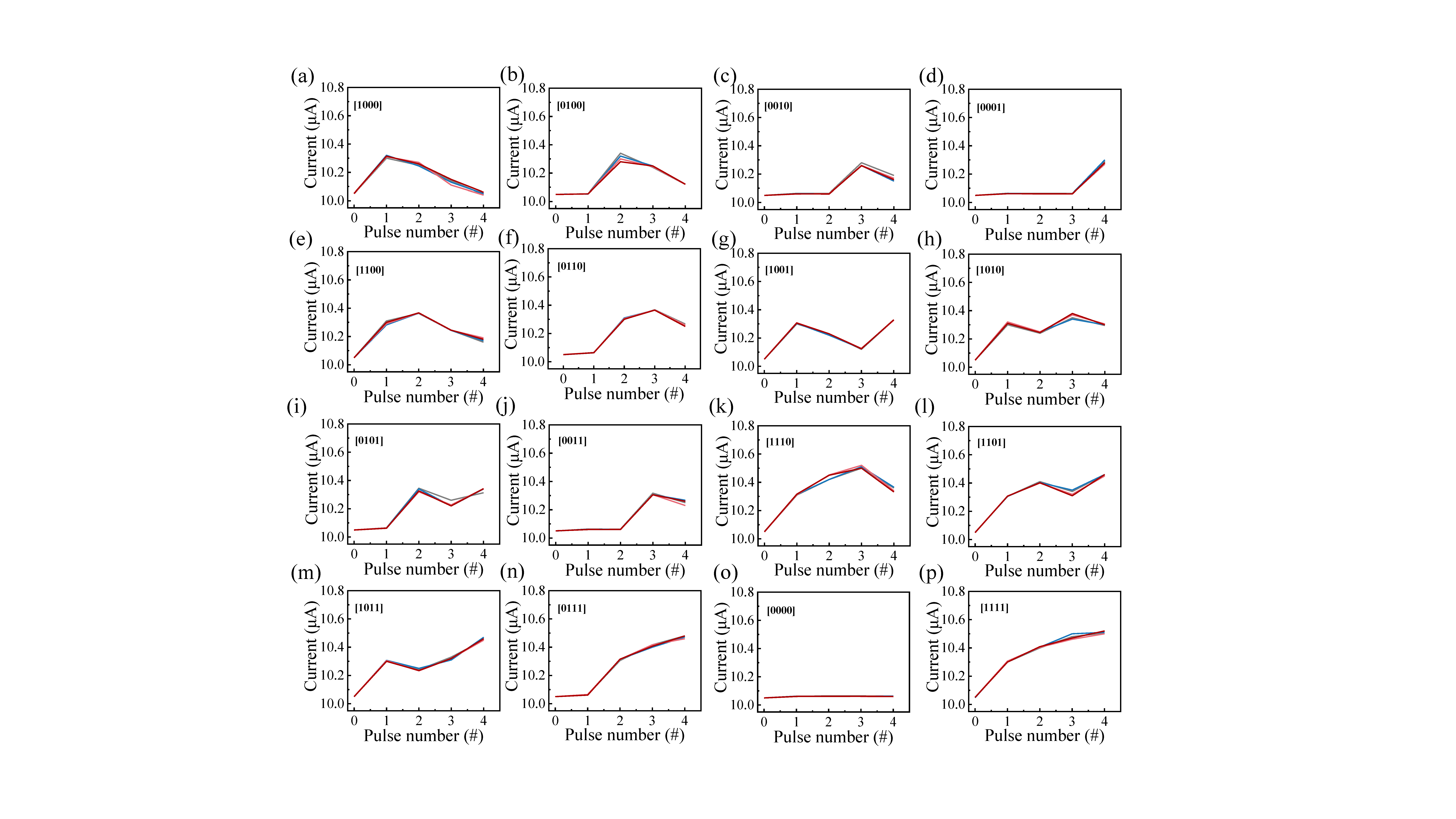


Figure R11 (a)-(p) Experimental read-current responses of Te-based optoelectronic memristor by electrical input signals.


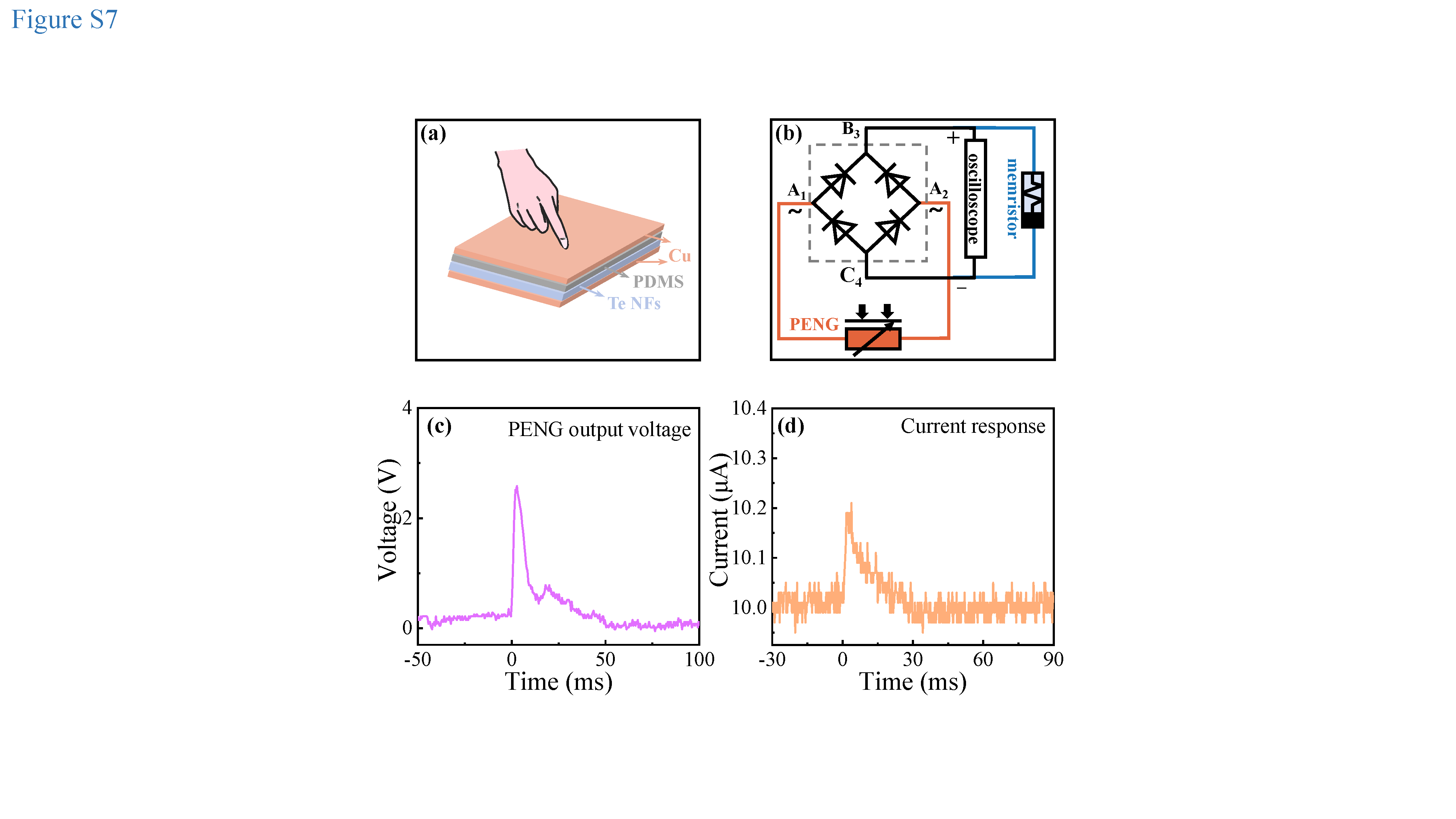


Figure S12. (a) PENG based on Te nanoflakes. (b) The equivalent circuit contains a memristor and PENG. (c) The open circuit voltage generated by the PENG. (d) The current response curves of the SPP Te-based memristor.

Along with photoconductivity and thermoelectricity, Te exhibits piezoelectric properties due to its non-centrosymmetric structure. We developed a piezoelectric nanogenerator (PENG) using multiple plate-like stacked Te nanoflakes instead of a single one in our manuscript. A low-temperature hydrothermal method was used to synthesize plate-like Te nanoflakes.^[1]^ Figure S12(a) shows a schematic illustration of PENG, which consists of a Cu/PDMS/Te nanoflakes/Cu sandwich structure. Herein, the thickness of the PDMS-coated Te nanoflakes and the Cu layers serving as the bottom and top electrodes is 500 μm and 50 nm, respectively. Furthermore, the output voltage generated by the PENG under the compressive force of 3 N and the current response of the SPP Te-based memristor were closely monitored via the equivalent circuit, as shown in Figures S12(b-d). The above results show the pressure-electrical conversion function for visual-haptic multimodal perception.


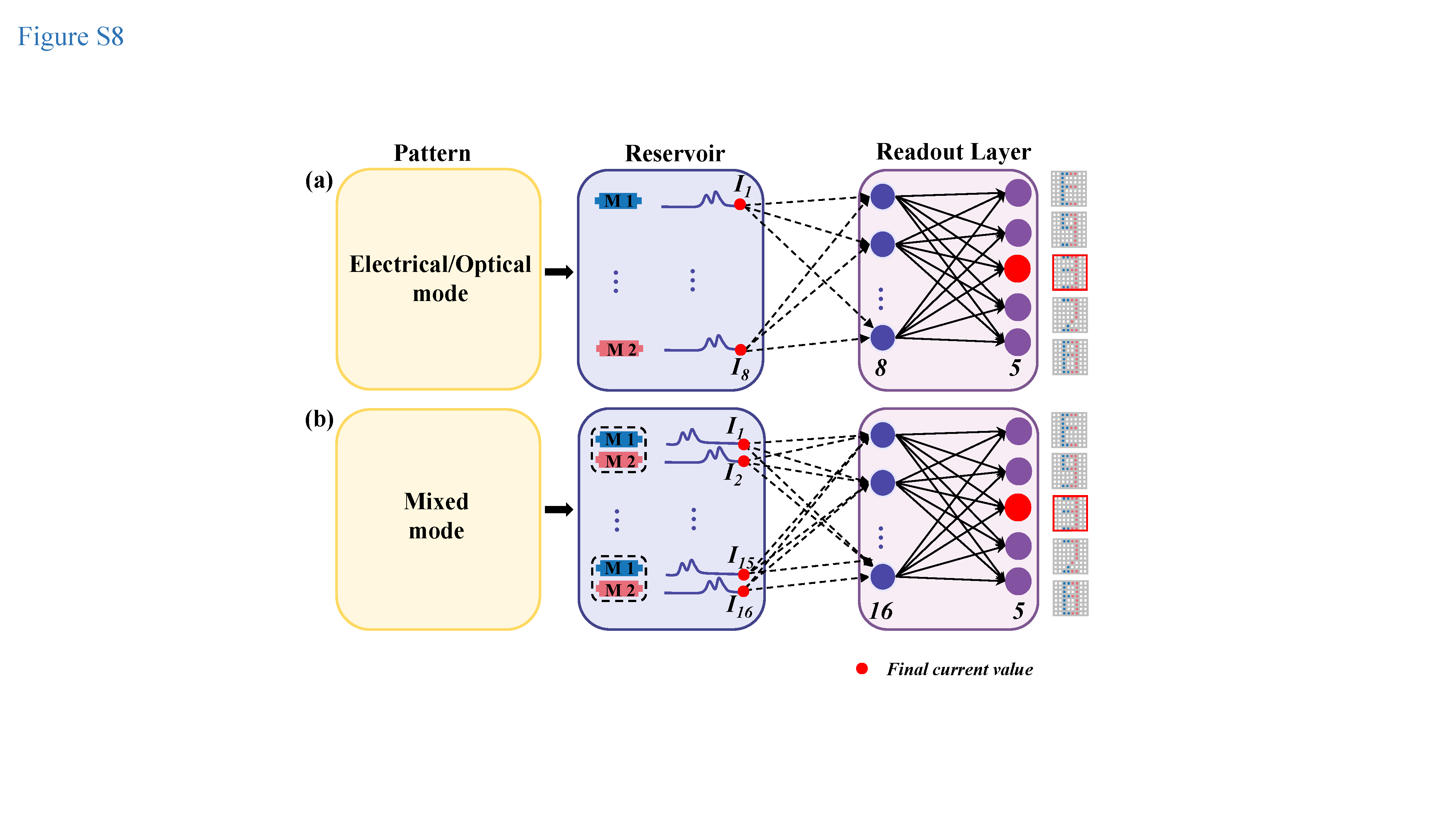


Figure S13.Schematic diagrams of the ANN for digital pattern classification under electrical/optical input and mixed input modes.

Figures 13(a) and (b) illustrate the schematic diagrams of an artificial neural network (ANN) designed for digital pattern recognition. For recognition tasks in only electrical or optical input mode, an 8×5 single-layer ANN was employed in this work, while a 16×5 single-layer ANN was used for mixed input recognition. Herein, the final current values (red dots) collected from the memristors are fed into the ANN for digital pattern recognition.


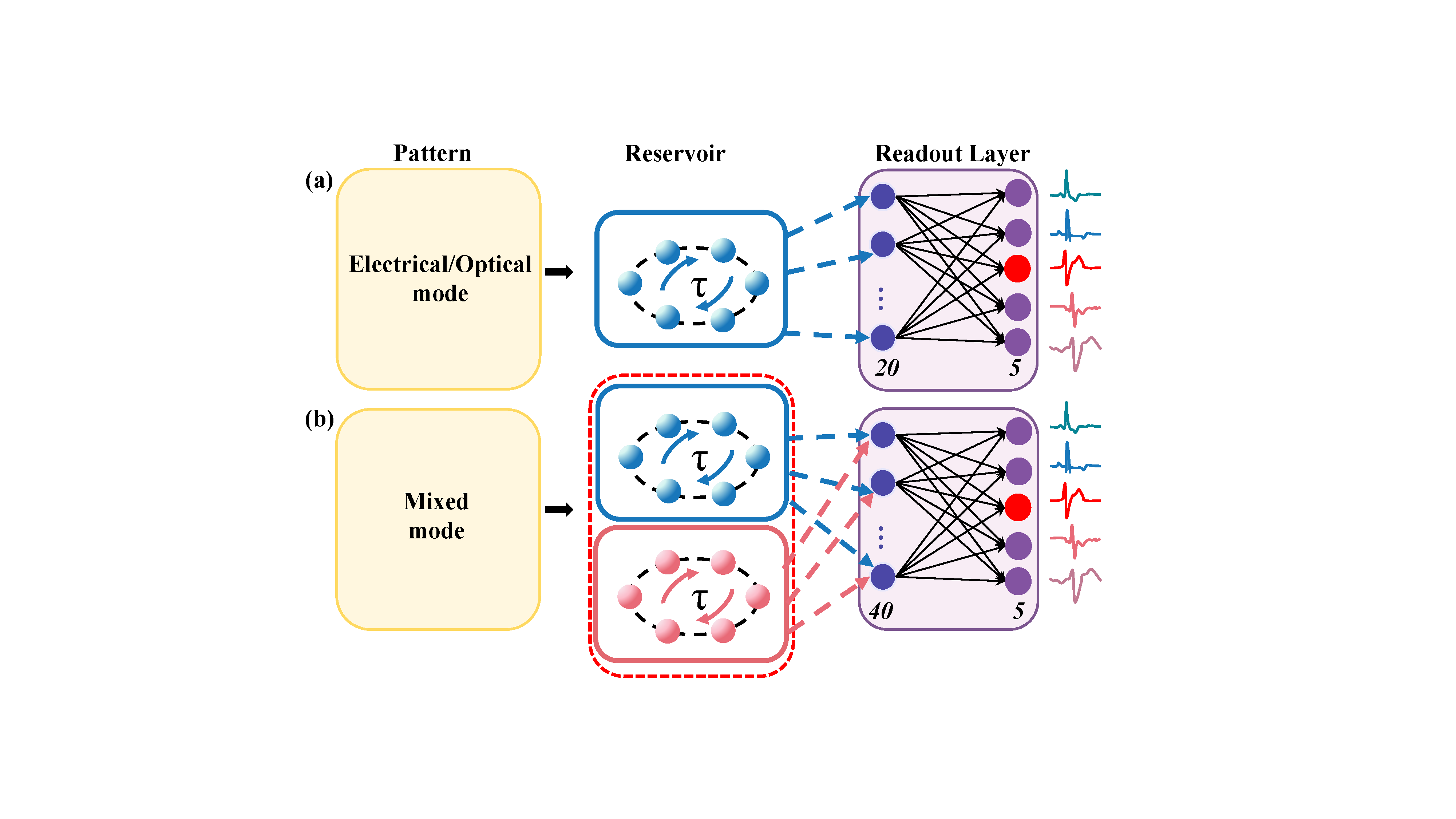


Figure S14. Schematic diagrams of the ANN for ECG pattern classification under electrical/optical input and mixed input modes.

For ECG pattern recognition tasks, the twenty virtual node current values (green pentagram) obtained after each sequence are inputted into an ANN for the purpose of pattern classification. A 20×5 single-layer ANN was employed in only electrical or optical input mode (Figure S14(a)) and a 40×5 single-layer ANN for mixed input recognition (Figure S14(b)).

References

[1] T. Il Lee, S. Lee, E. Lee, S. Sohn, Y. Lee, S. Lee, G. Moon, D. Kim, Y. S. Kim, J. M. Myoung, Z. L. Wang, *Adv. Mater.* **2013**, 25, 2920-2925.
